# Complex patterns on HEp-2 indirect immunofluorescence assay in a large sample referred for anti-cell autoantibodies detection

**DOI:** 10.3389/fimmu.2023.1256526

**Published:** 2024-01-12

**Authors:** Wilton Ferreira S. Santos, Ana Paula de Castro Cantuária, Daniele de Castro Félix, Natália Carvalho Guimarães, Igor Cabral Santos de Melo

**Affiliations:** ^1^ Immunology Division, Sabin Medicina and Health, Brasília, Brazil; ^2^ Long-term Care Unit, Hospital Geral de Fortaleza, Fortaleza, Ceará, Brazil

**Keywords:** antinuclear antibodies, autoantibodies, autoimmunity, HEp-2 cells, ANA patterns, indirect immunofluorescence

## Abstract

**Introduction:**

The combination of patterns is a frequent and challenging situation in the daily laboratory routine of autoantibodies testing using HEp-2 cells indirect immunofluorescence assay (HEp-2-IFA). Recently, the Brazilian Consensus on Autoantibodies (BCA) named these combinations as complex patterns (CPs) and organized them into 3 subtypes: multiple, mixed, and composite. This study aimed to describe the most frequent combinations of HEp-2-IIF patterns according to this new nomenclature.

**Methods:**

Routine HEp-2-IFA results reported in January and June 2017 were reviewed using the new BCA classification. Visual pattern recognition was performed by experts on HEp-2-IFA readings, using the International Consensus on Antinuclear Antibodies (ANA) Patterns (ICAP) and BCA recommendations.

**Results:**

54,990 serum samples from different patients were tested for ANA-HEp-2, and 11,478 (20.9%) were positive at a titer ≥ 1/80. Among these positive samples, 1,111 (9.7%) displayed CPs, divided into 95 different combinations. A higher proportion of CPs was observed in the pediatric age group. Multiple, mixed, and composite patterns were present in 85.3, 5.4, and 9.5% of the samples, respectively. In the multiple/mixed pattern group (n=1,005), double, triple, and quadruple combinations (ICAP/BCA codes) were observed in 97.7%, 2.2%, and 0.1%, respectively. The double nuclear pattern was the most prevalent combination observed (67.6%). The most common CPs registered were AC-4 (nuclear fine speckled) + AC-6,7 (nuclear discrete dots) (n=264); AC-2 (nuclear dense fine speckled) + AC-6,7 (n=201); AC-4+AC-8,9,10 (nucleolar) (n=129); and AC-3 (centromere)+AC-4 (n=124). All of these combinations were in the multiple subgroup.

**Conclusion:**

Almost 10% of positive results in the HEp-2 procedure displayed CPs. Among the 3 subtypes of CPs proposed, the multiple pattern was the most prevalent, especially in the pediatric population. The AC-4, AC-2, and AC-6,7 were the most prevalent single patterns observed in the combinations described in this study. There was a significant association between age and the prevalence of most combined patterns. The AC-4+AC-6,7 combination was the most prevalent complex pattern detected regardless of the age group. The AC-2+AC-6,7 was more prevalent in younger individuals. The concepts involved in the CPs definition should add value to the reading and interpretation of the HEp-2-IIF assay.

## Introduction

The Brazilian Consensus on Autoantibodies (BCA) recognizes 34 different positive staining patterns observed in the indirect immunofluorescence assay on HEp-2 cells (HEp-2-IFA). BCA-HEp-2 proposed the distribution of these patterns into 5 groups: nuclear, cytoplasmic, nucleolar, mitotic, and complex. The International Consensus on ANA Patterns (ICAP) recognizes 29 different positive patterns distributed into 3 groups: nuclear, cytoplasmic, and mitotic. Nucleolar staining is included in the nuclear group, and the complex patterns (CPs) are not depicted on the ICAP classification tree (1, 2).

Anti-cell (AC) alpha-numeric codes were ascribed for each HEp-2 fluorescence pattern by the ICAP initiative. Therefore, the 29 ICAP-positive patterns received codes ranging from AC-1 to AC-29. All ICAP codes have been adopted by the BCA, except one, AC-28, described as mitotic chromosomal. On the other hand, there are 5 patterns recognized by the BCA that are not addressed by the ICAP (2, 3).

The challenges in defining the fluorescence morphology from serum samples that present a combination of patterns have received the attention of the Brazilian consensus meeting participants since the II BCA in 2003. The terms “mixed pattern” or “composite pattern” were adopted to describe some situations where more than one cellular compartment is depicted due to the presence of a single antibody in the same sample or an overlap of patterns is observed due to the presence of more than one antibody (4).

This classification was updated at the VI BCA meeting that was held in September 2019 during the 36° Brazilian Congress of Rheumatology in Fortaleza-CE, Brazil. The group of CPs was introduced in the classification tree of BCA-HEp-2, thereby replacing the denomination of mixed patterns (1).

Based on the possible combinations of stained patterns and cell compartments distinguished by visual reading of HEp-2 cell slides, the CPs were organized into 3 subgroups: multiple, mixed, and composite. These subgroups were defined as follows: 1) multiple: a combination of patterns clearly identified on visual reading due to the presence of more than one autoantibody; 2) mixed: a mix of different patterns in the same cellular domain, not readily and identified at visual reading; and 3) composite: a combination of patterns due to the presence of a single autoantibody (1).

HEp-2 cells are considered an array for several target antigens. Serum samples that show reactivity against more than one antigen are not uncommon in daily laboratory routine. Under this circumstance, the correct interpretation of the HEp-2-IIF assay is challenging, requiring training and expertise (5–7).

Given the complexity and relevance of the CPs in daily laboratory practice, this study aimed to describe the most frequent pattern combinations observed on visual reading of HEp-2 cell slides in a large diagnostic medicine center in Brazil and to reclassify the mixed patterns recorded according to the new terminology proposed by VI BCA-HEp-2.

## Methods

### Study design

This is a retrospective and descriptive study in which the results of HEp-2-IFA carried out between January and June 2017 were reviewed. In this period, 57,265 consecutive serum samples from 54,990 patients were referred to the laboratory for routine autoantibody testing on HEp-2 cells. In cases where there was more than one serum sample from the same patient, only the first sample was included in the analysis. For each sample, gender and age were identified for further demographic analysis. It was not possible to obtain the clinical data of the participants. Patients and the public were not involved in the design, conduct, reporting, or dissemination plans of this research.

### Report review

All reports with mixed/composite patterns in the period of the study were reviewed. This review included the fluorescence patterns released, the titers defined for each antibody observed, and their respective ICAP/BAC codes. As recommended by the BCA, our report structure includes the description of the staining morphology of each cellular compartment/structure: nucleus, nucleolus, cytoplasm, mitotic apparatus, and metaphase plate. Internal observations that were helpful in the interpretation of reading procedures were also included in the analysis worksheet and consulted whenever necessary. After performing these procedures, the mixed patterns were reclassified according to the new BCA-HEp-2 proposal (1).

### HEp-2 indirect immunofluorescence testing

Anti-cell autoantibody tests were carried out on HEp-2 cell slides using the IIF technique according to the manufacturer’s instructions (*Euroimmun Medizinische Diagnostika*, Lübeck, Germany). A confirmatory test was performed using another manufacturer (INOVA Diagnostics, San Diego, CA, USA) whenever necessary. Double serial dilution of positive samples was performed for endpoint-titer determination. Titers ≥ 1/80 were reported as positive for any fluorescence patterns observed in either the nucleus, cytoplasm, or mitotic apparatus as recommended by the BCA (3). Visual pattern recognition was performed by 3 experts on HEp-2-IIF readings. Pattern definitions were established according to ICAP and BCA-HEp-2 recommendations.

### BCA and ICAP HEp-2-IIF pattern classification differences

On its decision tree, BCA-HEp-2 includes some patterns that are not recognized by the ICAP. These patterns received specific Brazilian Anti-Cell (BAC) codes, ranging from BAC-1 to BAC-5. The BAC-1, BAC-2, and BAC-5 are classified as composite CPs, while BAC-3 and BAC-4 represent elementary nuclear patterns. BAC-1 describes a nuclear fine speckled with nucleolar speckled pattern plus the decoration of the Nucleolar Organizing Regions (NOR) at the metaphase plate. BAC-2 describes a combination of nuclear coarse speckled, with nucleolar homogeneous and peripheral staining of the metaphase plate. BAC-5 presents a cytoplasmic dense fine speckled pattern with faint homogeneous staining in the nucleoli due to the presence of anti-P-ribosomal protein. BAC-3 and BAC-4 are defined as nuclear speckled *Quasi-*homogeneous and nuclear speckled reticular coarse patterns, respectively.

CENP-F-like (AC-14), NuMA-like (AC-26) and topoisomerase I-like (AC-29) are all patterns recognized by the ICAP that are included in the branch of the CPs on the Brazilian classification tree. Together with BAC-1, 2, and 5, they make up the composite subgroup of the CPs (1). BCA-HEp-2 does not recognize the ICAP AC-28 mitotic apparatus staining pattern. In the reports reviewed, some ICAP/BCA patterns were not detailed by the laboratory. The nucleolar homogeneous (AC-8), clumpy (AC-9), and speckled (AC-10) were reported in a grouped form as AC-8,9,10; the nuclear membrane continuous (AC-11) and dotted (AC-12) patterns were reported in a grouped form as AC-11,12; and the cytoplasmic fibrillar (AC-15), filamentous (AC-16) and segmental (AC-17) patterns were reported in a grouped form as AC-15,16,17. Likewise, the nuclear speckled with multiple (AC-6) and few discrete dots (AC-7) were reported as AC-6,7. Thus, although the BCA recognizes 34 positive patterns on HEp-2-IFA, due to the grouping of some patterns, only 27 were reported herein.

### Autoantibodies detection data review

Patient’s laboratory data reporting mixed patterns were reviewed to obtain information about specific autoantibodies testing in the samples assayed for HEp-2-IIF. Autoantibodies against DNA, Ro/SS-A, La/SS-B, Sm, U1-RNP, Jo-1, and Topoisomerase-I antigens are routinely tested in the laboratory if ordered by the clinician. In the period analyzed, anti-dsDNA antibodies were determined by IFI using *Crithidia lucilae* as substrate. Test positivity was considered for titles ≥1/10. Antibodies against the Extractable Nuclear Antigens (ENAs) encompassing Ro, La, Sm e RNP, Topoisomerase-I, and Jo-1 antigens were performed by fluorimetric enzyme-linked immunoassay (FEIA). Positivity and technical procedures for anti-ENAs testing were carried out according to the manufacturer’s instructions (Thermo Fisher Scientific, Inc., MA, USA).

### Statistical analysis

Categorical data were presented using frequency distributions and were analyzed using the chi-square test or Fisher’s exact test when indicated. The Normality of the data was verified using the Kolmogorov-Smirnov test. For quantitative variables, the Mann-Whitney test was used for comparisons between two groups, and the Kruskal-Wallis test was used for comparisons involving multiple groups. In the latter case, *post hoc* pairwise comparisons were performed using the Bonferroni procedure. Analyses were performed using SPSS 22 (Statistical Package for the Social Sciences, IBM, USA) for Mac and Epi Info™ for Windows version 7.2 CDC, USA. Statistical significance was indicated by α < 0.05.

### Ethics statement

This study was approved by the Research Ethics Committee of the *Centro Universitário de Brasília* (UNICEUB) – Protocol number: 3.466.204/2019. According to the terms of this approval, all patient data and information were anonymized and deidentified before analysis and the results were presented in a grouped way so that individual results were not possible to be identified. Based on these, the absence of written informed consent was accepted by the Ethics Committee and no specific consent procedure was required for this study.

## Results

During the 6 months studied herein, 54,990 different samples were tested for autoantibodies on HEp-2 cells, and 11,478 (20.9%) were positive at a titer ≥ 1/80. Of these, 1,111 (9.7%) displayed a combined staining pattern and were reclassified as CPs. The majority of the samples (n=10,367) were described using an ICAP/BCA single pattern.

### Demographic data

Of the 54,990 sera, 74.3% were from female patients. Among all positive results (n=11,478), the proportion of samples from females was 85.1%. This amount was quite similar to the proportion of females in the complex-pattern group, which was 86%.

The age range of all participants was 1-102 years old and the median age was 43 years old. The age range of the 11,478 HEp-2-IFA-positive patients was 1-97 years. HEp-2-IFA-positive results were separated into a single-pattern group (n=10,367) and a complex-pattern group (n=1,111). The median ages of the single-pattern and complex-pattern groups were 42 and 38 years old, respectively. Participants in the complex-pattern group were younger than those in the single-pattern group (Mann-Whitney test, *p* <.001).

### Combinations of patterns according to HEp-2 cells anatomy

The report structure used in our laboratory routine encompasses the description of the staining patterns in some cellular regions and structures: nucleus, nucleolus, cytoplasmic mitotic apparatus, and mitotic chromosome plate.

The patterns distribution of the 11,478 positive sera, according to cellular compartments was: nuclear (83.1%), cytoplasmic (4.0%), nucleolar (2.5%), and mitotic (0.7%). The combination of patterns or domains in the same sample (CPs) was observed in 9.7% of the patients.

Concerning the multiple and mixed subgroups, the nuclear region was involved in some of the reported combinations in 990 (98.5%) sera. Multiple/mixed patterns without nuclear staining were restricted to 15 out of all 1,005 samples. In these 1,005 samples, double (n=982), triple (n=22), and quadruple (n=1) combinations of patterns (ICAP/BCA codes) were observed in 97.7, 2.2, and 0.1%, respectively.

Initially, considering combinations involving 2 patterns, the double nuclear (2Nu) pattern was reported in 679 out of the 1,005 sera (2Nu=67.6%). One nuclear with nucleolar (Ncl) pattern was observed in 147 samples (1Nu+1Ncl=14.6%). A single nuclear pattern with a cytoplasmic (Cy) pattern was observed in 118 sera (1Nu+1Cy=11.7%), and one nuclear pattern with a mitotic (Mi) pattern was observed in 23 sera (1Nu+1Mi=2.3%). Double combinations without nuclear staining, including double cytoplasmic staining (2Cy), were observed in 9 samples (2Cy=0.9%). One cytoplasmic pattern with nucleolar staining was observed in 3 sera (1Cy+1Ncl=0.3%), and one cytoplasmic pattern with a mitotic pattern was observed in 3 reports (1Cy+1Mi=0.3%). In addition to these samples with double combinations involving the nucleus (n=967) and not involving the nucleus (n=15), 22 reports with triple pattern combinations and one sample with 4 ICAP/BCA patterns (2Nu+2Cy) were observed. The triple combinations comprised 3Nu (n=4); 2Nu+1Ncl (n=2); 2Nu+1Cy (n=8); 2Nu+1Mi (n=1); 1Nu+2Cy (n=1); 1Nu+1Ncl+1Cy (n=5); and 1Nu+1Cy+1Mi (n=1) (**Table 1**).

**Table 1:** Stained cellular domains in 1,005 serum samples depicting multiple and mixed patterns on HEp-2 cells indirect immunofluorescence assay (HEp-2-IFA).

Type of combination	Cellular Domains*	Frequency (n)	Frequency (%)
Double	2Nu	679	67.6
	1Nu+1Ncl	147	14.6
	1Nu+1Cy	118	11.7
	1Nu+1Mi	23	2.3
	2Cy	9	0.9
	1Cy+1Ncl	3	0.3
	1Cy+1Mi	3	0.3
Triple	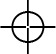	22	2.2
Quadruple	2Nu+2Cy	1	0.1
Total		1,005	100

*Nu, Nuclear; Ncl, Nucleolar; Cy, Cytoplasmic; Mi, Mitotic.

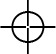
Triple combinations: 3Nu (n=4); 2Nu+1Ncl (n=2); 2Nu+1Cy (n=8); 2Nu+1Mi (n=1); 1Nu+2Cy (n=1); 1Nu+1Ncl+1Cy (n=5); 1Nu+1Cy+1Mi (n=1).

### Single patterns and CPs according to ICAP and BCA proposals

Among the 11,478 positive results, nuclear fine speckled (AC-4), nuclear dense fine speckled (AC-2), nuclear *quasi-*homogeneous (BAC-3), CPs (as a group), and nuclear homogeneous (AC-1) were the 5 most prevalent single patterns, and they were observed in 37.7%, 21.3%, 10%, 9.7% and 6.8% of the samples, respectively. These 5 patterns accounted for 85.5% of all HEp-2-IFA positive results. Besides BAC-3, BAC-4 (nuclear reticular coarse) was another pattern recognized only by the BCA observed among the positive HEp-2-IFA results. Its frequency as a single pattern was 0.9% and in combination with another ICA/BCA pattern was 1,2%. We observed only one description compatible with BAC-5 (cytoplasmic dense fine speckled plus nucleolar homogeneous). The presence of BAC-1 (nuclear fine speckled with nucleolar speckled pattern plus the decoration of the Nucleolar Organizing Regions) was not possible to define because, in 2017, the nucleolar fluorescence was not discriminated by the laboratory. Finally, we did not find any matching description for BAC-2 (nuclear coarse speckled plus nucleolar homogeneous).

We observed 95 different combinations of staining patterns in the 1,111 samples classified as CPs. From these, 81, 11, and 3 types of combinations were described in the multiple, mixed, and composite patterns, respectively. In the multiple/mixed subgroups (n=1,005), the AC-4 pattern was observed 644 times. The AC-6,7 pattern was present in 512 combinations. The AC-2 and AC-3 patterns were identified in 212 and 160 combinations, respectively. The vast majority of the AC-6,7 pattern was due to AC-7 (few nuclear dots); however, we did not separate these two patterns systematically, so it was not possible to determine accurately their prevalence rates.

According to the new BCA proposal, the complex-pattern group was divided into 3 subgroups: multiple, mixed, and composite. Considering the 1,111 samples classified as CPs, the percentage of multiple (n=948), mixed (n=57), and composite (n=106) observed was 85.3%, 5.1%, and 9.5%, respectively.

#### Pattern combinations in the multiple subgroup

The most common combinations of patterns observed in the multiple subgroup (n = 948) were AC-4+AC-6,7 (nuclear discrete dots) (n=260, 27.4%); AC-2+AC-6,7 (n=201, 21.2%); AC-4+AC-8,9,10 (nucleolar) (n=129, 13.6%); AC-3 (centromere) +AC-4 (n=124, 13.1%); and others (n=230, 24.3%). The “other group” included combinations between AC-4 and several cytoplasmic patterns (n=63); AC-6,7 with cytoplasmic patterns (n=16); AC-6,7 + BAC-3 (n=15); AC-1 + AC-5 (n=14); AC-4 + AC-5 (n=13); AC-4+BAC-4 (n=12); AC-4 with several mitotic patterns (n=11) and BAC-3 with several cytoplasmic staining patterns (n=11). The remaining multiple combinations appeared at a frequency of less than 1% (n ≤ 10) (n=75).

#### Pattern combinations in the mixed subgroup

The most prevalent combinations in the mixed subgroup (n = 57) were AC-1+AC-5 (nuclear coarse speckled) (n=14); AC-4+AC-5 (n=13); AC-4+BAC-4 (nuclear reticular coarse speckled) (n=12); AC-1+AC-4 (n=8); AC-3+AC-11,12 (n=4) and others (n=6). The other group comprised the following combinations: AC-1+BAC-4; AC-1+AC-13; AC-2+AC-11,12; AC-4+AC-11,12; BAC-3+BAC-4; BAC-3+AC-11,12; (one sample of each).

#### Pattern combinations in the composite subgroup

The most common composite patterns (n=106) observed herein were AC-26 (NuMA-like) (n=99), AC-29 (Topo I-like) (n=6) and AC-14 (CENP-F) (n=1). In this review, it was possible to identify one serum with a description compatible with BAC-5 (AC-19 + AC-8,9,10), classified as multiple, since the nucleolar decoration was not specified. No report compatible with BAC-1 and BAC-2 was identified. In 2 samples, it was reported the combination of NuMA-like with cytoplasmic patterns (AC-18 and AC-21). Both samples were classified as multiple. In one sample with a history of positive anti-dsDNA, it was observed the metaphase plate was homogeneously stained. But as the nuclear staining was registered as fine speckled, it was classified as composite rather than mixed.

### Single patterns and CPs prevalence according to age

The AC-4, AC-2, BAC-3, CPs, and AC-1 were the most prevalent patterns observed in 11,478 HEp-2-IFA-positive patients. For analyzing the prevalence of HEp-2-IIF elementary patterns and the CPs as a group according to age, the sample was divided into two groups: under 40-year-old (n=5,227) and over 40-year-old group (n=6,251). The prevalence of female sex in each group was 83.3% and 86.6% respectively.

The AC-4 and AC-2 patterns and the CPs as a group were more prevalent in individuals under 40 years old (*p* <.0001). The prevalence ratios (RP) for these patterns in this age group were higher for CPs and AC-2 than AC-4. The BAC-3, AC-1, and the “other patterns” are more common in individuals ≥ 40 years old (*p* <.001). The RP varied from 0.56-0.77, indicating a lower risk for younger individuals to present these patterns. These data adjusted for sex are presented in **Table 2**.

**Table 2:** Frequency (%) of the most prevalent ICAP/BCA single patterns and the complex patterns as a group in 11,478 HEp-2-IFA positive samples, according to two age groups.

HEp-2-IFA Patterns^⋆^	Group 1 < 40 years old (n=5,227) Female=83.3%		Group 2 ≥ 40 years old (n=6,251) Female=86.6%		PR*	95%CI*	χ2^✦^	*p*-value^✧^
	n	%	n	%				
AC-4	2,093	40.0	2,232	35.7	1.12	1.07-1.17	21.4	*p* < .0001
AC-2	1,297	24,8	1,153	18.4	1.19	1.15-1.24	79.2	*p* < .0001
BAC-3	370	7.1	782	12.5	0.57	0.51-0.64	88.9	*p* < .0001
Complex Patterns 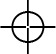	582	11.2	529	8.5	1.32	1.18-1.48	23.4	*p* < .0001
AC-1	307	5.9	476	7.6	0.78	0.68-0.90	11.8	*p* = .0006
Others	578	11.0	1,079	17.3	0.64	0.58-0.70	89.0	*p* < .0001

ICAP, International Consensus on Antinuclear Antibodies (ANA) Patterns;

BCA, Brazilian Consensus on Autoantibodies;

IFA, Indirect Immunofluorescence Assay.

⋆ AC-4: Nuclear Fine Speckled; AC-2: Nuclear Dense Fine Speckled; BAC-3: Brazilian Anti-Cell Code-3: Nuclear *Quasi*-homogeneous; AC-1: Nuclear Homogeneous.

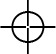
Complex patterns as a group.

* PR, Prevalence Ratio; CI, Confidence Interval.

✦ Chi-square statistic value: χ2, df=1, n=11,478

✧ Significance level for α = 0.05

**Figure 1:**
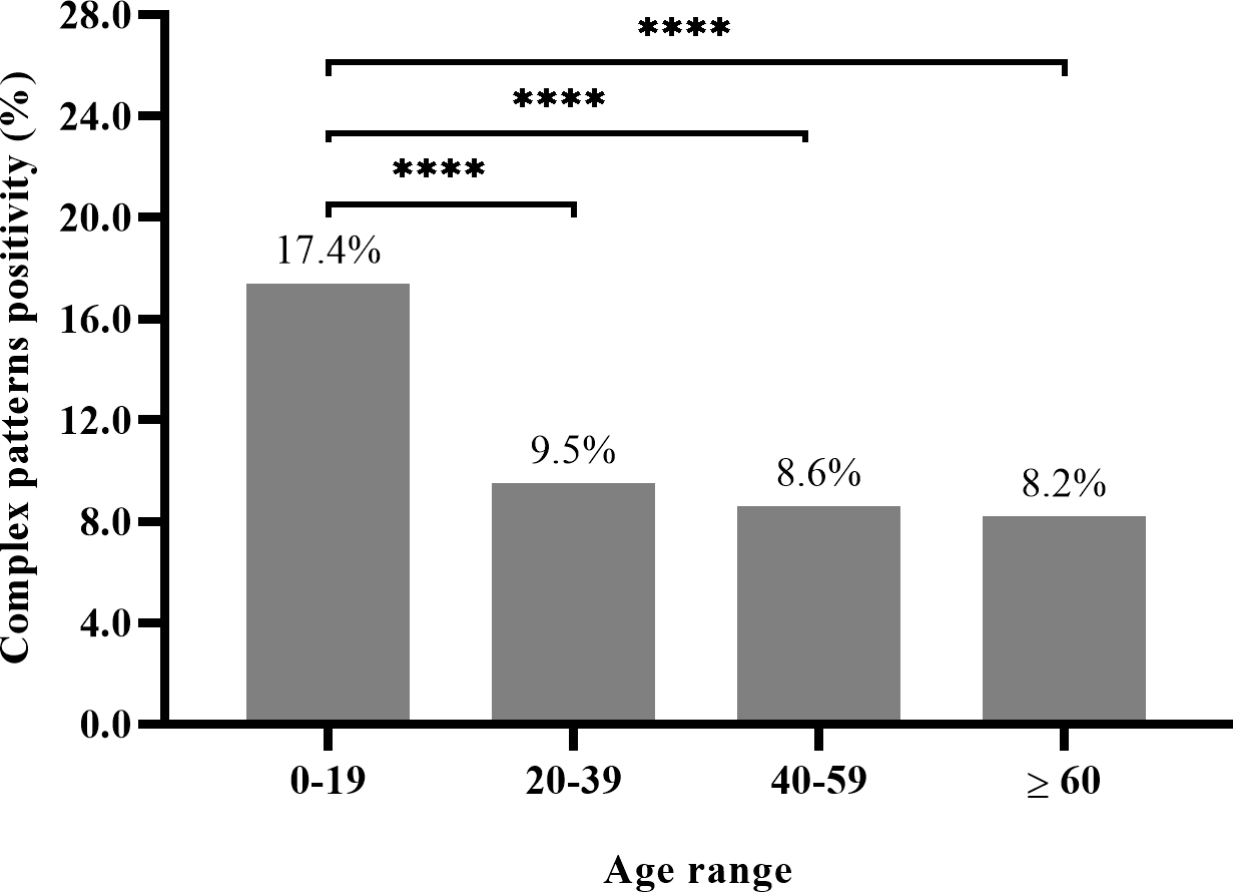
Complex patterns prevalence in 11,478 HEp-2-IFA-positive samples, based on the four age groups: pediatric (0-19), young adults (20-39), adults (40-59) and elderly (≥60 years old). (Chi-square test for differences between the 0- to 19-year-group and each one of the other groups with p = .0002, after Bonferroni correction were represented by ****). For the pediatric group (n=184) X the other groups (n=931) - analysis: X2= 99.4, Prevalence Ratio=2.15 (95%CI:1.85-2.49).

For additional analysis of the association between the prevalence of CPs and age, the sample was divided into 4 age groups: pediatric (0-19 years old, n=184), young adults (20-39 years old, n=398), adults (40-59 years old, n=348), and elderly (≥ 60 years old, n=185). The prevalence of CPs in each of these age groups was 17.4%, 9.5%, 8.6%, and 8.2%, respectively. A higher prevalence of CPs was observed in the 0- to 19-year-old group (Chi-square test, *p* = .0002) (**Figure 1**). In the pediatric group, CPs were the third most common pattern observed (17.4%), following the AC-4 (39.1%) and the AC-2 (24.7%) patterns.

The prevalence of positive ANA-HEp-2 results, regardless of the pattern, in the pediatric group was 23.2% (1058/4570). To better explore these data, we divided the ANA-positive pediatric group into 2 subgroups: children: 0-9 years old (n=258) and adolescents: 10-19 years old (n=800). A higher prevalence of autoantibodies as detected on HEp-2-IIF was observed in the adolescent group (24.3%) when compared to the children group (20.2%) (χ2 = 8.7, df=1, *p* = .003). The prevalence of ANA-HEp-2 positive results in the 11,478 ANA-HEp-2 positive samples, considering the other age ranges was 20.2%, 20.1%, and 22.8% for the young adults, adults, and elderly participants, respectively.

### Complex patterns combinations X age groups

**Figure 2:**
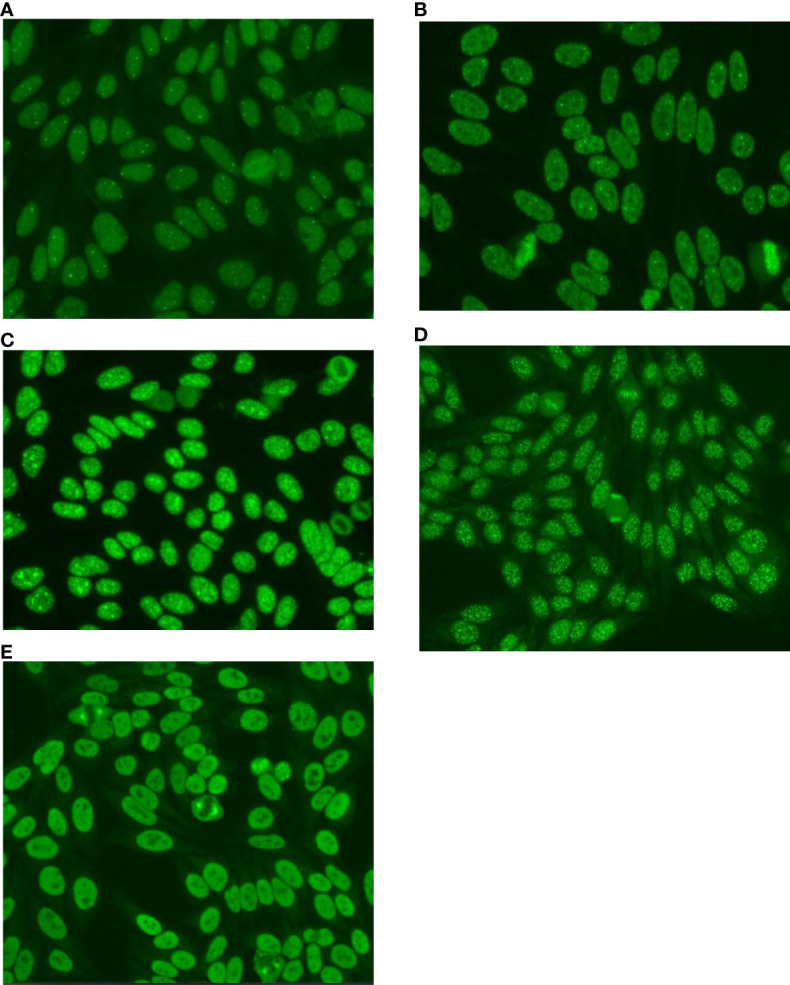
The most prevalent Complex Patterns in 1,111 HEp-2-IFA-positive samples, according to the Brazilian Consensus on Autoantibodies on HEp-2 cells (BCA) proposal. **(A)** - Multiple Pattern: AC-4+AC-7 - Nuclear fine speckled plus few discrete nuclear dots. **(B)** - Multiple Pattern: AC-2+AC-7 - Nuclear dense fine speckled plus few discrete nuclear dots. **(C)** - Multiple Pattern: AC-4+AC-8 - Nuclear fine speckled plus nucleolar homogeneous. **(D)** - Multiple Pattern: AC-4+AC-3 - Nuclear fine speckled plus nuclear centromere. **(E)** - Composite Pattern: AC-26 - NuMA-like. Nuclear fine speckled with fluorescence of the mitotic apparatus.

The five most prevalent combinations of patterns observed in the 1,111 samples with CPs were: AC-4+AC-6,7 (23.4%); AC-2+AC-6,7 (18.3%); AC-4+AC-8,9,10 (11.6%); AC-3+AC-4 (11.2%) and AC-26 (8.9%). Other pattern combinations were present in 26.6% of the patients. These five most prevalent CPs are illustrated in **Figure 2**.

Similar to what was done with single patterns, for the analysis of the association between the prevalence of CPs subtypes and age, the whole sample was divided into two groups: under 40 years old (n=582) and over 40 years old (n=529). The prevalence of female sex in each group was 82.3% and 89.9% respectively.

The results showed that AC-4+AC-6,7 and AC-2+AC-6,7 were more prevalent in the younger individuals (<40 years old). The chance of positivity for these combinations was almost double in this group of patients (prevalence ratio: 1,7-1,87). Combinations with the nucleolar pattern and the AC-26 pattern were more prevalent in individuals over 40 years old. The low prevalence ratios (0.56 and 0.48) observed indicated a lower chance of finding these combinations in people under 40 years old (*p* <.0001 for both CPs). The “other patterns” were more prevalent in individuals over 40 years old, but it was especially more prevalent in elderly people over 60 years of age, where it reached a proportion of 44.3%. No difference in prevalence was observed between the age groups for the AC-4+AC-3 combination. These data adjusted for sex are presented in **Table 3**.

**Table 3:** Frequency (%) of the most prevalent Complex Patterns in 1,111 consecutive HEp-2-IFA positive samples, according to two age groups.

HEp-2-IFA Patterns^⋆^	Group 1 < 40 years old (n=582) Female=82.3%		Group 2 ≥ 40 years old (n=529) Female=89.9%		PR*	95%CI*	χ2^✦^	*p*-value^✧^
	n	%	n	%				
AC-4+AC-6,7	175	30.1	85	16.1	1.81	1.81-2.28	26.6	*p* < .0001
AC-2+AC-6,7	133	22.9	70	13.4	1.71	1.31-2.22	15.7	*p* < .0001
AC-4+AC-8,9,10	49	8.4	80	15.1	0.56	0.40-0.78	11.4	*p* = .0007
AC-4+AC-3	72	12.4	52	9.8	1.28	0.91-1.79	1.76	*p* = .18 / NS
AC-26	34	5.8	65	12.3	0.48	0.31-0.70	13.8	*p* = .0002
Others	119	20.5	177	33.3	0.63	0.52-0.77	19.9	*p* < .0001

IFA, Indirect Immunofluorescence Assay.

^⋆^AC-4: Nuclear Fine Speckled; AC-2: Nuclear Dense Fine Speckled; AC-8,9.10: Nucleolar; AC-3: Nuclear Centromere; AC-26: NuMA-like.

* PR, Prevalence Ratio; CI, Confidence Interval.

✦ Chi-square statistic value: *χ*2, df=1, n=1,111.

NS, Not Significant

✧ Significance level for α = 0.05

For additional analysis of the association between the prevalence of some subtypes of CPs and age, the sample was divided into the same 4 age groups described previously: pediatric, young adults, adults, and elderly.

Considering the 3 subtypes of CPs proposed by the BCA, multiple patterns were observed in 177 of 184 sera (96.2%) of the pediatric group (0-19 years old, n=184). The proportion of mixed and composite patterns in this group was only 1.6% and 2.2% respectively. Taking the other groups together (n=927), the proportion of multiple, mixed, and composite was 83.2%, 5.8%, and 11%, respectively. The higher prevalence of multiple patterns in the pediatric group was significant (χ^2^ = 20.8, df=1, *p* <.001).

The prevalence of the AC-4+AC-6,7 combination in the pediatric group (44.0%, n=184) was higher when compared to the other groups (19.3%, n=927) (χ^2^ = 52.3, df=1, *p* <.0001). In elderly patients (≥ 60 years old, n=185), the “other patterns” accounted for almost half of the CPs (44.3%). The prevalence of these patterns in the elderly group was highly significant when compared to the prevalence in the group under 60 years old (n=926) (χ2 = 35.9, df=1, *p* <.0001). The combination of nuclear fine speckled plus nucleolar was the second most prevalent (18.4%) in this group. By the way, the prevalence of this combination increased with age (*p* = .0007) (**Table 3**). Similarly, the proportion of “other patterns” gradually increased with age.

### Antigenic specificities of the autoantibodies detected

From the 1,111 samples that depicted a combined immunofluorescence pattern, 242 (21.8%) were tested simultaneously for the presence of specific autoantibodies. Positive results for any of the requested antibodies were observed in 56 samples (23.1%). In only 20 (8.3%) samples more than one autoantibody was detected.

The most frequent specific autoantibodies ordered together with the ANA-HEp-2 were: anti-dsDNA (n=175), anti-Ro (n=155), anti-La (n=136), anti-Sm (n=107) anti-U1-RNP (86); anti-Topoisomerase-I (n=40) and anti-Jo-1 (n=8). The positivity rates for these autoantibodies in this study were: anti-dsDNA=6.9%; anti-Ro=24.5%; anti-La=9.6%; anti-Sm=10.3%; anti-RNP=7% and anti-Topoisomerase-I= 17.5%. None of the samples tested for anti-Jo-1 were positive.

The 36 samples with only one autoantibody detected, presented a nuclear pattern associated with another immunofluorescence pattern due to an antigen not tested or unknown. The most frequent autoantibodies identified in this subgroup were anti-Ro (n=18), anti-dsDNA (n=6), anti-Topoisomerase-I (n=6), anti-Sm (n=4), and anti-RNP (n=2). The fluorescence pattern corresponding to the autoantibody detected was not identified in 10 samples (27.8%). The ICAP patterns related to Ro (AC-4) and RNP (AC-5) antigens were not identified in these samples.

In the 20 samples with more than one autoantibody detected, we observed the presence of 2 (n=14), 3 (n=5), and 4 autoantibodies (n=1). All of them are due to reactivities against dsDNA and ENA profile (Ro, La, Sm, U1-RNP). The fluorescence pattern expected was detected in 14 samples. In the 6 (30%) remaining samples, at least one expected pattern was not observed. All of these samples were classified as mixed patterns according to the new VI BCA proposal.

### Titers of each pattern in the combinations evaluated

Considering the highest titration observed in each combination, titers of 1/80, 1/160, 1/320, 1/640, 1/1280, and ≥1/2560 were observed in 4.3, 19.5, 43.7, 22.6, 9.3, and 0.6% of the 1,005 sera with multiple and mixed patterns, respectively. Serum dilution procedures can help identify fluorescence patterns present in samples with two or more autoantibodies (multiple and mixed patterns), provided they are present at different titers. In fact, in these subgroups, the titers of the patterns were different in only 204 sera (20.3%). For the most frequent patterns (e.g., AC-4+AC-6,7; AC-2+AC-6,7; AC-4+AC-8,9,10; and AC-3+AC-4), both autoantibodies, in each combination, were present in the same titer in 83.3% of the situations. Different titers were observed in 18.6% (49/264) of the combinations with AC-4+AC-6,7 and in 14.4% (29/201) of the combinations with AC-5-AC-6,7. In these groups, the titer of the AC-6,7 was higher than that of the AC-4 and AC-2 in 93.9% and 79.3% of the cases, respectively. For the AC-4+AC-8,9,10 and AC-3+AC-4 patterns, different titers were observed in 14.7% (19/129) and 18.5% (23/124), respectively. In these cases, the nucleolar and centromere staining were stronger than that of the AC-4, at 84.2 and 95.6%, respectively.

## Discussion

National and international standardization initiatives have yielded valuable contributions to the reading and interpretation of HEp-2-IIF staining patterns, thereby paving the way for the standardization of reports and the improvement of the interpretation of the HEp-2-IFA results. The Brazilian HEp-2-IFA consensus movement started in 2001, and in its sixth edition, efforts were directed towards the organization of the mixed patterns, designated by the VI BCA-HEp-2 as CPs. CPs are described in the literature as mixed, multiple, composite, combined, overlapping, miscellaneous, or simultaneous patterns. To the best of our knowledge, this is the first article to specifically address this issue.

Based on the VI BCA recommendations, the reports of 54,990 HEp-2-IIF assays issued in the first half of 2017 were reviewed and all samples classified previously as mixed patterns were reclassified as CPs, considering the three subtypes proposed: multiple, mixed, and composite (1). Among the 11,478 HEp-2-IFA-positive results, we identified 1,111 (9.7%) samples with CPs, according to the VI BCA proposition. The CPs, as a group, were the fourth most common pattern observed. The AC-4, AC-2, and BAC-3 were the 3 most frequent single patterns identified in all positive HEp-2-IFA samples. In the literature, the prevalence of mixed patterns varies from 3.5 to 28.6% (5, 8–14). This wide range of positivity may be explained by differences in sampling, reasons for requesting the exam, as well as the degree of adherence to international recommendations on ANA testing and reporting. Prado et al., in a patient-based study with 269 systemic lupus erythematosus (SLE) patients, found that the multiple overlapping patterns were the third most frequent, present in 21.2% of the SLE patients. Only AC-1 (29.3%) and AC-4 (28.6%) single patterns were more prevalent than the combined HEp-2 patterns (14). In a hospital-based study involving 3,960 consecutive patients, following the AC-4, the mixed patterns were the second most prevalent pattern in 866 HEp-2-positive patients from several medical specialties (9). Reports with multiple/mixed ANA patterns account for an important part of the daily routine workup on autoantibody diagnostics.

Some combinations of patterns play a relevant role in the investigation and evaluation of autoimmune diseases (1, 5, 15). Wei et al., in a patient-based study with 4,583 subjects (3,510 with Systemic Autoimmune Rheumatic Diseases - SARD and 1,073 Health Individuals), found ANA positivity in 78.7% of SARD patients. AC-4, AC-1, AC-5, AC-8-9, and mixed patterns were the most frequent results in the SARD group. According to these authors, mixed patterns provided hints for systemic sclerosis and systemic lupus erythematosus (SLE) (12). Soldani and Hadalwar found 13.5% mixed patterns in 280 ANA-positive samples from 650 patients. Speckled (17.85%) and homogeneous (8.59%) nuclear patterns were the most frequent single patterns reported (10). The combinations of reticular and speckled cytoplasmic patterns with nuclear patterns such as AC-4, AC-1, and AC-3 were described in an expressive proportion of patients with autoimmune liver diseases (16). A distinctive combination of patterns, involving the concomitant presence of cytoplasmic and nuclear patterns, such as AC-21, AC-3, AC-6, and AC-12 can provide relevant diagnostic and prognostic information in patients with primary biliary cholangitis (15).

Regarding the HEp-2 cells anatomy and the topographic distribution of the patterns found in our study, the proportion of nuclear, nucleolar, cytoplasmic, mitotic, and mixed patterns reported was 83.1%, 2.5%, 4.0%, 0.7%, and 9.7%, respectively. These distributions of the HEp-2 patterns, regarding its cellular compartments, are in line with other publications, except for the cytoplasmic domain that appears overrepresented in some studies (9, 11, 17). According to some authors, the prevalence of cytoplasmic patterns increases with age (17). This trend was observed in this study and will be discussed later. The proportion of the single and mixed patterns according to HEp-2 cell domains in different studies is presented in **Table 4**. As in the ICAP proposal, the nucleolar patterns are arranged within the nuclear group, the truly nuclear combinations of patterns account for 82.2% of the CPs. The double nuclear (67.6%) and the nuclear plus nucleolar combination (14.6%) were by far the most common combinations observed (**Table 1**). Recognizing multiple antibody reactivities in the same cellular compartment can be a challenging task and requires training (6, 7).

**Table 4:** Single and complex patterns on HEp-2 cells, according to cellular domains reported by different authors.

Author/year [Reference]	Country	Sample size n	HEp-2+ n (%)	Single Patterns – Cellular domains (%)*				Mixed Patterns (%)
				Nu	Ncl	Cy	Mi	
Dellavance et al., 2005 (8)	Brazil	30,728	13,641 (44.4)	91	2	2	1	5
Laurino et al., 2009 (9)	Brazil	3,960	866 (21.7)	72.9	5.8	10.1	0.1	11
Satoh et al., 2012 (17)	USA	4,754	670 (14.1)	84.6	6.1	21.8	–	✦
Sodani & Hawaldar, 2018 (10)	India	650	280 (43.1)	58.2	7.9	5.0	0.3	28.6
Gupta et al., 2020 (11)	India	538	179 (33.2)	59.8	8.9	18.9	8.4	3.9
Wei et al., 2020 (12)	China	4,583	1,893 (41.3)	86.8	5.3	3.5	0.9	3.5
Krzemien et al., 2022 (13)	Poland	1,731	260 (15.0)	74.4	2.6	20.6	2.5	50^✧^
Santos et al., Present study 2024	Brazil	54,990	11,478 (20.9)	83.1	2.5	4.0	0.7	9.7

*Nu, Nuclear; Ncl, Nucleolar; Cy, Cytoplasmic; Mi, Mitotic.

✦ Mixed patterns are mentioned by the authors but not specified.

✧ Krzemien et al., 2022 (13) the samples analyzed by the authors comprised 1,731 sera presenting with ANA patterns considered “rare” by the researchers. The percentage 50% is related to the “rare” samples. The number (%) of mixed patterns considering all the ANA-HEp-2-positive samples is not possible to define in the article.

### ICAP and BCA patterns classification differences

The BCA-HEp-2 incorporated the category “mixed patterns” into its classification tree in 2003. In its 6^th^ edition (1), BCA named this category as CPs and divided it into three subgroups: multiple, mixed, and composite. ICAP uses the terminology mixed or multiple to describe the situation in which more than one staining pattern is seen on the HEp-2-IIF assay in the same sample. The proposition of creating the composite patterns as a separate category did not reach consensus in the ICAP meetings (18, 19). Unlike the BCA, the multiple/mixed patterns do not figure in the ICAP decision tree. Some other differences between these initiatives will be discussed below.

The Brazilian consensus distinguishes a higher number of HEp-2-IFA patterns than ICAP. This favors a higher number of pattern combinations. The BCA recognizes all the patterns proposed by the ICAP but one, the AC-28. On the other hand, there are five patterns recognized solely by the BCA, identified with specific Brazilian Anti-Cell codes (BAC) ranging from BAC-1 to BAC-5. The BAC-1, BAC-2, and BAC-5 codes describe a particular combination of patterns due to the presence of a single autoantibody and are classified as composite CPs. The BAC-1 pattern is a combination of nuclear and nucleolar staining patterns, associated with the presence of anti-RNA polymerase I/II antibodies. The BAC-2 code refers to the nuclear speckled and nucleolar homogeneous combination associated with the presence of anti-Ku antibodies. The BAC-5 is a combination of cytoplasmic dense fine speckled plus a nucleolar homogeneous, due to the presence of anti-ribosomal-P proteins. The BAC-3 (*quasi-*homogeneous) and BAC-4 (reticular coarse) are nuclear elementary patterns placed within the speckled group (1, 3).

**Figure 3:**
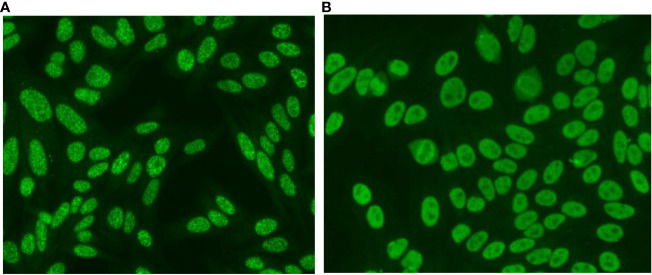
Subdivision of the nuclear fine speckled pattern (AC-4) into AC-4a and AC-4b patterns. **(A)** AC-4a: a myriad of discrete nuclear speckled in interphase cells. Nucleoli and mitotic chromosomes are not stained. **(B)** AC-4b: plain nuclear fine speckled. equal-sized fine speckles uniformly distributed throughout the nucleus. Nucleoli and mitotic chromosomes are not stained.

Besides these specific BCA patterns, in the last consensus meeting, the BCA proposed splitting the nuclear fine speckled pattern (AC-4) into AC-4a and AC-4b, for an optimal report (1). The AC-4a is described as a myriad of tiny discrete nuclear speckles observed in interphase cells with negative metaphase chromatin plate and is associated with the presence of Anti-SS-A/Ro60 antibodies (1, 4). The AC-4b is described as a plain fine speckled pattern, with multiple antigenic specificities, including Mi-2, TIF1γ, Ku, and RNApol II/III (1, 20–23). Representative images of these two patterns are shown in **Figure 3**. The acknowledgment of the AC-4 variations is gaining worldwide acceptance (20, 24) and its addition to the ICAP decision tree will be subject of discussion in the next ICAP meeting (19). We believe that keeping the nuclear discrete fine speckled pattern associated with the anti-Ro60 antibody within the AC-4 ICAP pattern was a good decision, considering the historical relationship that this autoantibody established with this pattern historically built over the last 5 decades. The description of the BCA patterns not recognized or not incorporated into the ICAP classification system, together with its possible correspondent ICAP codes, target antigens, and clinical relevance are presented in **Table 5**. Information on all ICAP patterns and their clinical relevance is available on the ICAP website (www.anapatterns.org).

**Table 5:** HEp-2-IFA – Immunofluorescence patterns recognized by the Brazilian Consensus on Autoantibodies (BCA) but not incorporated into the classification tree of the International Consensus on ANA Patterns (ICAP) – patterns description, target antigens, and clinical relevance.

BAC Code	BAC Classification tree	Correspondent ICAP Code(s)	Pattern description	Target antigens	Clinical relevance 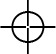
BAC-1	Complex Composite	AC-4+AC-10	Nuclear plain fine speckled. Nucleolar speckled. Metaphase Plate with discrete, bright dots.	RNA Polymerase I and III (22)	SSc with difuse cutaneous disease.
BAC-2	Complex Composite	AC-4+AC-8 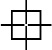	“*Fine speckled nucleoplasmic ANA decorating the interphase* *nuclei as well as the nucleoli*” (23) 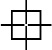 .	Ku	SSc-AIM and SLE-SSc-AIM overlap syndromes
BAC-3	Nuclear Speckled *Quasi*-homogeneous	AC-XX* (Not recognized by ICAP).	Thin speckled nuclear fluorescence. Metaphase Plate with same pattern.	Multiple antigenic specificities	Unknown
BAC-4	Nuclear Speckled reticular coarse	AC-5	large nuclear speckles arranged in a netwise configuration across the nucleus (1)	Nuclear matrix associated hnRNP	Unknown
BAC-5	Complex composite	AC-19+AC-8	Cytoplasmic dense fine speckled to homogeneous. Nucleolar homogeneous.	Ribosomal-P phosphoproteins (P0, P1, P2, C22 RibP peptide).	SLE
AC-4a	Subdivision of AC-4: Discrete fine speckled	AC-4	myriad of discrete nuclear speckles	Ro60	SLE, SjS, SCLE, NLE, congenital heart block
AC-4b	Subdivision of AC-4: Plain fine speckled	AC-4	“*uniform distribution of equal-sized fine speckles in the nucleoplasm*” (20)	Mi-2, TIF1γ,✧ Ku, RNApol II/III	AIM, SSc, SSc-AIM overlap

HEp-2-IFA, HEp-2 cells Indirect Immunofluorescence Assay.

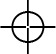
SLE, systemic lupus erythematosus; SSc, systemic sclerosis; SjS, Sjögren’s syndrome; SCLE, subacute cutaneous lupus erythematosus; NLE, neonatal lupus erythematosus; AIM, autoimmune inflammatory myopathy.

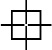
BCA describes the nucleoplasmic staining of the BAC-2, associated with anti-Ku antibodies as coarse speckled. ICAP and other authors describe it as nuclear fine speckled (20, 21, 23).

* AC-XX: ICAP code for unknown pattern. Misinterpretation with AC-1 or AC-2 may occur.

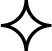
 TIF, transcription intermediary factor; RNApol, RNA polymerase II/III.

### Single and complex patterns prevalence according to the age groups

The AC-4 (37.7%), AC-2 (21.3%), and BAC-3 (10%) were the three most prevalent elementary ICAP/BCA codes observed in the 11,478 positive-HEp2-IIF assays. Together they represent almost 70% of all positive results. Except for the BAC-3 pattern, recognized solely by the BCA, our findings are similar to those obtained by different authors from different parts of the world (8, 10–13). We observed a higher prevalence ratio for AC-4 and mainly for AC-2 in the participants under 40 years old. The main target antigen associated with the classic AC-2 pattern is the anti-DFS70. The higher prevalence of the AC-2 pattern due to monospecific sera for anti-DFS70 antibodies in younger individuals is well established in the literature (25–28). Conversely, BAC-3 was more prevalent in individuals over 40 years of age. The BAC-3 pattern was especially more prevalent in elderly people (≥ 60 years old). The percentage of the BAC-3 pattern in the participants under and over 60 years old was 8.6% and 15.9%, respectively. This difference was highly significant (χ2 = 106.5, df=1, *p* <.0001) (data not shown).

In this study, we found a higher HEp-2-IFA positivity in children and adolescents (0-19 years) (HEp-2-IFA positivity=23.2%). Analyzing the results in children (0-9 years) and adolescents (10-19 years) separately, we observed a higher prevalence of anti-HEp-2 cell autoantibodies in adolescents (24.3%) when compared to children aged 0-9 years (20.1%). Thus, the higher prevalence of ANA in the pediatric group observed herein was due to the positivity of the test in the adolescent group. Sperotto et al., and Dinse et al. (29, 30), reported an increasing prevalence of autoantibodies during puberty, mainly in females. In a population study involving a large sample evaluated longitudinally, Dinse et al., reported a dramatic increase in ANA prevalence in adolescents aged 12-19 years. For the authors, the potential explanations for this finding include changes in perinatal or early-life exposures. Infections or other types of exposures during developmentally sensitive periods may lead to immune dysregulation (29). In another study involving 261 subjects aged 8-13 years, Speroto et al. (30), reported persistence and increased ANA titers in individuals transitioning from the prepubertal to the pubertal stage. ANAs were found more frequently in pubertal females than in males. Conflicting data in the literature can be exemplified by the research conducted by Hilario et al. (31), that showed a higher prevalence of autoantibodies in children aged 5-10 years in a study involving 214 healthy Brazilian children and adolescents aged 6 months to 20 years. In our study, the higher proportion of HEp-2 positive results in those aged 10-19 years was also followed by an expressive increase in the proportion of female subjects from 58.3% (0-9 years) to 75.7% (10-19 years). Sex hormones play an important role in modulating the immune response. Genetics, cytokine profiles, and other factors enhance this gender bias toward autoimmunity (30, 32). Therefore, an increased unspecific immune response in puberty, resulting in increased autoantibody production, isolated or in combination, might explain the higher prevalence of HEp-2-IFA positivity and indirectly explain the higher prevalence of CPs in this age group. The higher prevalence of autoantibodies in females is discussed elsewhere (33). The nuclear speckled, homogeneous, and nucleolar are the most common ANA patterns described in the pediatric population (30, 31, 34, 35). In our study, AC-4, AC-2, and CPs were the 3 most prevalent patterns observed in this age group and together accounted for 81.2% of all positive results. Mixed patterns are described as rare in children. The most reported pattern combinations comprise the associations between the 3 previously mentioned elementary patterns AC-1, AC-4, and AC-8,9,10 (31, 34). In our research, the most prevalent combinations seen in the pediatric group were the AC-4+AC-6,7 (44.0%) and AC-2+AC-6,7 (23.4%). Both prevalence rates were highly significant. We did not find similar report in the literature.

Considering the whole sample, the most common CPs identified were: AC-4+AC-6,7; AC-2+AC-6,7; AC-4+AC-8,9,10; AC-3+AC-4 and AC-26. As observed for the AC-4 and AC-2 single patterns, the combinations of these patterns with the discrete nuclear dots (AC-6,7) were more prevalent in the pediatric group and young adults. For the combined pattern AC-4+AC-8,9,10 and AC-26, a higher prevalence in the age group ≥ 40 years old was significant. The prevalence of different combinations increased with age. We observed that the proportion of “other patterns combinations” in elderly individuals reached 44.3%. As observed by Satoh et al., cytoplasmic patterns increased with age (17). In our study, the rate of positivity of cytoplasmic patterns increased progressively: 1.2%, 2.3%, 4.7%, and 7.4% in pediatric, young adult, adults, and elderly patients, respectively. ranges defined in this study. Cytoplasmic patterns increased with age. Therefore, the proportion of combinations involving the cytoplasmic domain was higher in individuals ≥ 60 years of age.

In the literature, the mixed patterns are cited without details about the patterns involved in the combinations. We identified reports of combinations containing mainly the patterns: nuclear speckled, nuclear homogeneous, nucleolar, and cytoplasmic (10, 31, 34) or citing combinations of stained cellular domains and the proportion of mixed patterns without specifying the patterns identified (11, 12, 17). We believe this is the first article that presents the mixed patterns specifying its compositions using an internationally standardized nomenclature.

### The prevalence of the AC-6,7 and BAC-3 patterns in the CPs identified

Among all 11,478 positive patterns, AC-4, AC-2, and BAC-3 were the most frequent elementary patterns observed. However, in the complex-pattern group, AC-4, AC-6,7, and AC-2 were the most common patterns identified in the several combinations described. The AC-6,7 was frequently seen in combinations with other patterns, mainly with AC-4 and AC-2 patterns. Considering that this pattern is not among the most prevalent elementary patterns in the sample, its high frequency among the complex pattern samples was somewhat surprising. Reviewing the notes made in the laboratory reports, we found that the vast majority of the samples reported as AC-6,7 were due to the presence of “few nuclear dots” (AC-7). The peculiar fluorescence of the AC-6,7 pattern, characterized by the presence of few (1 to 6 dots) or multiple (6-20 dots) in the nucleus of the interphase cells, makes it easier to recognize it, even in the concomitant presence of another pattern in the same cellular domain. This could be one possible reason why the AC-6,7 pattern was so prevalent in the multiple pattern subgroup described herein. Both patterns have defined antigenic associations. The most known antigen related to the AC-7 pattern is p80 coilin. This antigen accumulates in high concentration in nuclear structures observed near the nucleoli named Cajal bodies. Anti-p80 coilin antibodies are considered specific markers of Cajal bodies. Besides p80 coilin, Cajal bodies contain small nuclear ribonucleoproteins (snRNPs) and the survival of motor neuron proteins (SMN), proteins related to spinal muscular atrophy. There are no defined clinical conditions associated with the presence of anti-p80 coilin antibodies (21, 36, 37). The multiple nuclear dots pattern (AC-6) is associated with anti-Sp100, anti-MJ/NXP-2, and anti-PML, being useful in the diagnosis of diseases such as primary biliary cholangitis and autoimmune myopathy (1, 15, 21). Therefore, making the distinction between AC-6 and AC-7 has recognized clinical value and is recommended by both initiatives (1, 21). Goto et al., evaluated the relationship between anti-p80 coilin autoantibodies and anti-DFS70 autoantibodies (AC-2 pattern) (36). These authors showed that the occurrence of anti-p80 coilin was strongly associated with the presence of anti-DFS70 antibodies. According to these authors, anti-p80 coilin and anti-DFS70 were described more often in females and additionally, younger individuals were more likely to be positive for anti-p80 coilin. These observations are in line with our results that showed a higher prevalence of AC-2+AC-6,7 combination in individuals < 40 years old (*p* <.0001). Furthermore, Tomic Sremec et al., in a populational study involving 10,955 individuals, found 4,107 ANA-HEp-2 positive sera. Among them, they identified 1,743 (42.4%) samples with rare immunofluorescence patterns. The authors defined as “rare” all patterns present in ≲ 3% of all samples. The AC-6 (n=58) and AC-7 (n=49) patterns represented 2.6% of all ANA-positive samples. A total of 22 ICAP patterns were considered rare, according to the authors’ criteria. The simultaneous presence of other patterns was observed in more than 80% of the samples that depicted AC-6 and AC-7 patterns. Among the 22 ICAP patterns considered “rare”, AC-6 and AC-7 were the patterns that most often appeared together with other patterns (38). In our study, AC-6,7 was the second most common single pattern found in association with another ICAP pattern.

**Figure 4:**
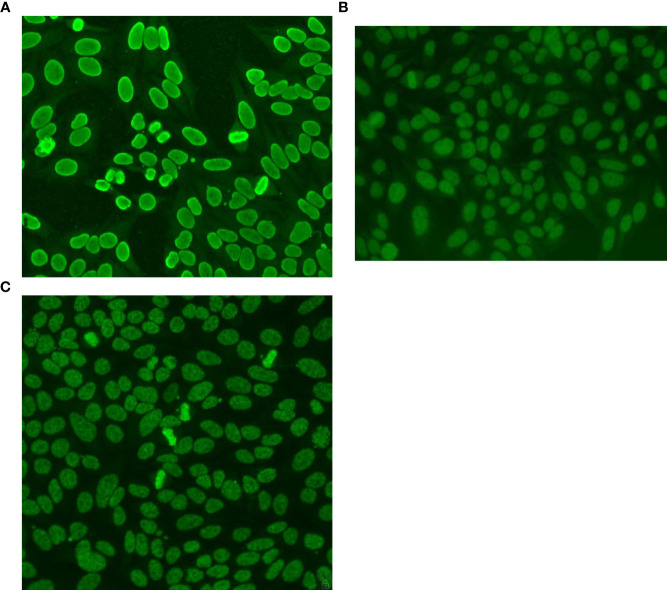
HEp-2 Patterns – Nuclear Homogeneous and Speckled patterns with stained chromatin plate. **(A)** AC-1 – Nuclear Homogeneous: uniform diffuse fluorescence covering the entire nucleus. The chromatin plate of metaphase cells depicts a smooth, hyaline, fluorescence pattern. The nucleoli are often covered by the nuclear fluorescence. The anti-dsDNA antibodies, carried out by ELISA, were positive. **(B)** BAC-3 – Nuclear Speckled Quasi-Homogeneous: Speckled fluorescence, approaching the homogeneous texture throughout the nucleus. The chromatin plate depicts the same pattern. The nucleoli are not stained. The anti-dsDNA antibodies, carried out by ELISA, were negative. **(C)** AC-2 - Nuclear Dense Fine Specked: Fine speckles throughout the interphase nucleus, with heterogeneity in size, and brightness. Denser and looser areas of speckles are seen throughout the nucleus. The chromatin plate is strongly stained. The nucleoli are not stained. The anti-DFS70/LEDGF antibodies, carried out by ELISA, were positive.

Unlike the discrete nuclear pattern that was overrepresented in the combinations observed herein, we found that BAC-3 was underrepresented in the CPs group. We expected more combinations involving the BAC-3 pattern since it was the third most frequent single pattern in the HEp-2-positive samples. We believe that this was due to a misinterpretation of the pattern when it was in combination with another nuclear pattern. The BAC-3 presents an intermediary morphological feature between the AC-1 and AC-2 patterns. Its reactivity reflects a heterogeneous profile of autoantibodies and unlike the AC-2 pattern, its presence is associated with several clinical conditions (39, 40). Besides, the BAC-3 positive samples usually do not show high reactivity on HEp-2 cells and the presence of another antibody in higher concentration may make its accurate identification extremely difficult (7, 41). The AC-1, AC-2 and BAC-3 immunofluorescence patterns are presented in **Figure 4**. The recognition of even isolated anti-DFS70 by IIF is challenging. Samples with mixed patterns containing traditional ANA patterns associated with AC-2 are incorrectly identified even by experienced laboratory technicians (6, 7). Recently a novel HEp-2-IIF pattern, similar to AC-2, has been described and named DFS-like or “pseudo-DFS” pattern. This pattern depicts a uniform brightness of the nuclear speckles without the heterogeneity in size and distribution of the speckles seen in the classical AC-2 pattern. The chromatin plate keeps the same features of the nucleoplasm (42). Considering the description, this novel pattern is also very similar to the BAC-3 pattern. Like the AC-4 pattern, maybe an umbrella terminology, bringing together all these patterns, would be the simplest solution to put into practice. Then all patterns morphologic related to the “DFS family” would be identified with the AC code and an identity for the subpattern (e.g., AC-2a, AC-2b…). The accurate interpretation of this “umbrella pattern” would imply confirmation of anti-DFS70 reactivity and other follow-up tests, according to clinical suspicion. The BAC-3 pattern was introduced in the Brazilian decision tree in the IV BCA, which occurred in 2013. Almost a decade later, its importance is recognized by only 23% of Brazilian rheumatologists (43).

### Autoantibodies specificities

The antigenic specificity of the anti-cell autoantibodies was tested in 242 (21.8%) samples. A restricted number of specific autoantibodies were available for testing and the majority of the requests comprised basic anti-ENA profile (Ro, La, Sm, U1-RNP) and anti-dsDNA. A total of 56 samples in 242 sera were positive for at least one antibody and only 20 (8.2%) sera presented more than one reactivity. We do not adopt reflex testing or any algorithm for autoantibody testing based on the ANA pattern. The follow-up testing for specific autoantibodies must be ordered by the clinician (44).

Anti-dsDNA, anti-Ro/SSA anti-La/SSB, and anti-Sm were the most requested antibodies, but the most prevalent were anti-Ro/SSA, anti-Sm, and anti-La. In general, anti-ENA antibodies are identified as the most prevalent in ANA-positive patients. There are some reports in the literature of higher prevalence of anti-Ro (3.9%) (17), anti-DFS70 (3.2%), and anti-nDNA (1.7%) (45) in ANA-positive individuals from population-based studies. In patient-based studies the positivity rate varies from 19-44% depending on the diseases included, the methodology of testing, and the number of autoantibodies tested. Anti-ENA and anti-nDNA are routinely included in the requests. Among these autoantibodies, the anti-Ro is usually the most prevalent antibody detected (10, 46, 47).

In this study, we analyzed whether the reported combined patterns corresponded to those expected considering the autoantibodies identified. The simultaneous presence of two or more autoantibodies was detected in only 20 samples. We observed that in 6 of these samples, one or more expected patterns were not correctly identified. All of them were classified as mixed, according to the VI BCA proposal. According to BCA, the mixed subgroup of CPs comprises a mixture of “different patterns in the same cellular domain, not readily and clearly identified at visual reading”. For these cases, the BCA recommends reporting the description “nuclear mixed pattern” without the AC codes (1). We did not find any reference about the positivity of specific antibodies in samples depicting mixed patterns to make a comparison with our findings.

Mitotic cell analysis and sample dilution can help in the pattern identification process. Metaphase plate staining characteristics facilitate the recognition of certain ICAP/BCA codes. Patterns such as AC-1, AC-2, AC-3, BAC-1, BAC-3, and AC-29 present a staining pattern in the metaphase plate that helps to distinguish them from each other, from the AC-4 pattern and from other patterns where the mitotic chromatin is not stained. Sample dilution procedures allow the distinction between overlapping patterns that are present in different titrations (39). In our analysis, only 204 (20.3%) samples in the multiple and mixed subgroups (n=1,005) showed differences in the pattern’s titers. We believe this number could be higher. The presence of one pattern in moderate or high titles may have discouraged the search for an end-titer definition of the other pattern. The added value for the end-titer definition of extremely high titer samples is still a matter of debate; however, end-point titration is recommended by international organizations and the BCA, especially in situations of overlapping patterns (39, 48). In samples with discordant titers, the fluorescence intensity of the discrete nuclear dots (AC-6,7), centromere (AC-3), and nucleolar pattern (AC-8,9,10) prevailed over the other patterns.

### Study limitations

The retrospective design of the study may have had an impact on the number of CPs classified as mixed since it is difficult to judge retrospectively if and how much the pattern presented in the combination observed was different from the expected original fluorescence pattern. Retrospection may also have influenced the number of samples with discrepant titles, which was likely underestimated in our study. The lack of clinical data did not allow the CPs of clinical relevance analysis.

### Final considerations

ICAP and BCA recommendations promote considerable improvements in the daily routine workup of autoantibodies testing on HEp-2 cells. The benefits of the nomenclature standardization process are recognized worldwide. All patterns have a nominal, numerical, and group identity, which appears to be sufficiently informative for the decision-making process in the clinical setting. The complex pattern proposal brings relevant concepts to the reading and interpretation routine of the HEp-2-IIF testing. These concepts undoubtedly will favor the analysis and interpretation of fluorescence patterns in the HEp-2 cells, promoting improvements in the analytical phase of the test. The inclusion of information about subtypes of complex patterns in the reporting phase of the HEp-2-IIF assay probably will not add value to the diagnostic decision-making process.

## Conclusions

Almost 10% of positive results in routine HEp-2-IIF testing displayed a combination of fluorescence patterns classified as CPs. CPs as a group were among the fourth most frequently encountered patterns in the HEp-2-IFA laboratory routine. Considering the HEp-2 cell anatomy, the double nuclear pattern was by far the most observed cellular domain combination. Among the 3 subtypes of CPs proposed, the multiple pattern was the most prevalent, especially in the pediatric population. The AC-4, AC-2, and AC-6,7 were the most prevalent single patterns observed in the combinations described in this study. There was a significant association between age and the prevalence of most pattern combinations identified. The AC-4+AC-6,7 combination was the most prevalent complex pattern detected regardless of the age group. The concepts involved in the CPs subtype definition should add value to the reading and interpretation of the HEp-2-IIF assay.

## Data availability statement

The raw data supporting the conclusions of this article will be made available by the authors, without undue reservation.

## Ethics statement

This study involving humans was approved by the Research Ethics Committee of the Centro Universitário de Brasília (UNICEUB). The studies were conducted in accordance with the local legislation and institutional requirements. The ethics committee/institutional review board waived the requirement of written informed consent for participation from the participants or the participants’ legal guardians/next of kin because it was a retrospective study, involving the analysis of the results of Antinuclear Antibodies Assays, carried out in the first semester of 2017. The study reviewed the results of 54,990 tests. We presented the research protocol to the ethics committee and request the waiver of written informed consent. The ethics committee analyzed our project and our request and was in favor of carrying out the research as long as some requirements were met to guarantee the anonymity of participants.

## Author contributions

WS: Conceptualization, Data curation, Formal Analysis, Investigation, Methodology, Project administration, Supervision, Validation, Writing – original draft, Writing – review & editing. AC: Data curation, Formal Analysis, Investigation, Methodology, Writing – original draft, Writing – review & editing. DF: Data curation, Formal Analysis, Investigation, Methodology, Writing – original draft, Writing – review & editing. NG: Data curation, Formal Analysis, Investigation, Methodology, Writing – original draft, Writing – review & editing. IM: Data curation, Formal Analysis, Investigation, Methodology, Software, Writing – original draft, Writing – review & editing.
